# Identification of Key Signaling Pathways and Genes in Eosinophilic Asthma and Neutrophilic Asthma by Weighted Gene Co-Expression Network Analysis

**DOI:** 10.3389/fmolb.2022.805570

**Published:** 2022-02-02

**Authors:** Gongqi Chen, Dian Chen, Yuchen Feng, Wenliang Wu, Jiali Gao, Chenli Chang, Shengchong Chen, Guohua Zhen

**Affiliations:** ^1^ Division of Respiratory and Critical Care Medicine, Department of Internal Medicine, Tongji Hospital, Tongji Medical College, Huazhong University of Science and Technology, Wuhan, China; ^2^ Key Laboratory of Respiratory Diseases, National Health Commission of People’s Republic of China, National Clinical Research Center for Respiratory Diseases, Wuhan, China

**Keywords:** eosinophilic asthma, neutrophilic asthma, key genes, bioinformatics, weight gene co-expression network analysis

## Abstract

**Background:** Asthma is a heterogeneous disease with different subtypes including eosinophilic asthma (EA) and neutrophilic asthma (NA). However, the mechanisms underlying the difference between the two subtypes are not fully understood.

**Methods:** Microarray datasets (GSE45111 and GSE137268) were acquired from Gene Expression Omnibus (GEO) database. Differentially expressed genes (DEGs) in induced sputum between EA (*n* = 24) and NA (*n* = 15) were identified by “Limma” package. Gene Ontology (GO) and Kyoto Encyclopedia of Genes and Genomes (KEGG) pathway enrichment analyses and Gene set enrichment analysis (GSEA) were used to explore potential signaling pathways. Weighted gene co-expression network analysis (WGCNA) were performed to identify the key genes that were strongly associated with EA and NA.

**Results:** A total of 282 DEGs were identified in induced sputum of NA patients compared with EA patients. In GO and KEGG pathway analyses, DEGs were enriched in positive regulation of cytokine production, and cytokine-cytokine receptor interaction. The results of GSEA showed that ribosome, Parkinson’s disease, and oxidative phosphorylation were positively correlated with EA while toll-like receptor signaling pathway, primary immunodeficiency, and NOD-like receptor signaling pathway were positively correlated with NA. Using WGCNA analysis, we identified a set of genes significantly associated NA including *IRFG*, *IRF1*, *STAT1*, *IFIH1*, *IFIT3*, *GBP1*, *GBP5*, *IFIT2*, *CXCL9,* and *CXCL11*.

**Conclusion:** We identified potential signaling pathways and key genes involved in the pathogenesis of the asthma subsets, especially in neutrophilic asthma.

## Introduction

Asthma is a chronic airway inflammatory disease characterized by airway hyperresponsiveness, reversible airflow limitation, mucus overproduction and airway wall remodeling (King-Biggs, 2019; Gao et al., 2020). Asthma affects more than 300 million people worldwide with approximately 250,000 deaths per year (Scherzer and Grayson, 2018; Ijaz et al., 2019).

According to the differential counts of sputum inflammatory cells, asthma can be divided into four subtypes: eosinophilic asthma (EA), neutrophilic asthma (NA), mixed granulocytic asthma, and paucigranulocytic asthma (Tliba and Panettieri, 2019). EA and NA have attracted our attention on account of their differences in clinical features and therapeutic effects. EA is defined by having more than 3% of eosinophils in the sputum and with a variable severity (Erle and Sheppard, 2014; Schleich et al., 2016). Type 2 immune response plays an important role in EA. NA is defined by having more than 61% of sputum neutrophils and mostly occurs in more severe asthma (Pelaia et al., 2015; Taylor et al., 2018). Inhaled corticosteroids (ICS) is effective with airway eosinophilic inflammation, whereas neutrophilic asthma has poor response for ICS (Green et al., 2002; Jayaram et al., 2006). Although Th2 and Th17 signaling pathways are implicated in EA and NA, respectively, the underlying mechanism distinguishes these two subtypes remains unclear (Boonpiyathad et al., 2019).

Recently, bioinformatic methods have been widely applied to identify the robust differentially expressed genes (DEGs) and signaling pathways in a variety of diseases (Zeng et al., 2019). Weighted gene co-expression network analysis (WGCNA) is a widely used method in building co-expression pairwise correlation matrices (Xu et al., 2020). There are several studies on EA and NA which focus on DEGs screening, while the interconnection between the involved genes and different subtypes of asthma has not been investigated (Baines et al., 2014; Sánchez-Ovando et al., 2021).

In this study, we integrated two microarray datasets including EA and NA patients from gene expression omnibus (GEO) database. Gene Ontology (GO), Kyoto Encyclopedia of Genes and Genomes (KEGG) enrichment analyses, and Gene set enrichment analysis (GSEA) were used to identify the potential mechanisms that distinguish between EA and NA. In order to explore the relation between gene modules and asthma subtypes, WGCNA was performed and two modules were recognized to be positively related to EA and NA groups. Finally, we sorted out a series of genes based on gene significance (GS), module membership (MM) and the protein-protein interaction (PPI) network, which might play major roles in the pathogenesis in EA and NA.

## Materials and Methods

### Microarray Datasets Acquisition

We obtained microarray datasets from GEO (http://www.ncbi.nlm.nih.gov/geo) utilizing the getGEO function of the GEOquery package (version 2.58.0) in R software (version 4.0.4). We adopted (eosinophilic asthma) AND (neutrophilic asthma) as the search strategy and five microarray data were obtained. The following eligibility criteria were used to include or exclude datasets and samples: 1) Expression type is expression profiling by array; 2) Sample type is induced sputum; 3) Subjects are non-severe and steroid-naïve. GSE4511 (Baines et al., 2014) and GSE137368 were extracted from the GEO database. Both platforms were GPL6104. We obtained 15 EA samples and 11 NA samples in GSE45111 and 9 EA samples and 4 NA samples in GSE137268.

### Data Processing and Differentially Expressed Genes Identification

We considered consolidating two datasets since both used the same platforms. Data processing included data consolidation, batch normalization and ID conversion. The merged data were processed via ComBat normalization in sva package (version 3.38.0) in R software based on the classical Bayesian analysis to remove batch effects (Leek et al., 2012). We next identified DEGs between EA and NA group by an empirical Bayes method based on limma package (version 3.46.0) in R software (*p* < 0.05, |logFC|>0.5) (Ritchie et al., 2015). Volcano plot and heatmap were plotted by ggplot2 (version 3.3.3) and pheatmap (version 1.0.12) package, respectively.

### Functional and Pathway Enrichment Analyses

The GO and KEGG pathway enrichment analyses of DEGs were analyzed and visualized in clusterProfiler package (version 3.18.1) in R software (Yu et al., 2012). The Metascape website (http://metascape.org), which is an online analysis tool integrated with multiple ontology sources, was implemented to conduct GO and KEGG analyses of gene modules selected by WGCNA (Zhou et al., 2019).

### Gene Set Enrichment Analysis

GSEA analysis was performed using GSEA software (version 4.1.0) (Subramanian et al., 2005). KEGG was selected as the gene sets database. The gene set was deemed to significantly enriched with alpha or *p* values < 5% and false discovery rate (FDR) < 25% for each analysis, which was performed 1,000 times for each analysis. The parameter of GSEA are as following: The parameter “Collapse data set to gene symbols” is set to “false”. The parameter “Permutation type” is set to “phenotype”. The parameter “Enrichment statistic” is set to “weighted”, while the parameter “Metric for ranking genes” is set to “Singal2Noise”.

### Weighted Gene Co-Expression Network Analysis

We implemented WGCNA by the WGCNA package in the R software (Langfelder and Horvath, 2008). We selected top 5,000 median absolute deviation (MAD) genes to construct the representation matrix and the appropriate power parameter was decided by pickSoftThreshold function. We used hierarchical clustering to identify modules of highly interconnected genes on the basis of their connectivity and covariance coefficients. The heatmap was plotted to reflect the relationships between each module and subtypes of asthma. The modules were constructed with the threshold value of the module dendrogram of 0.25, and a minimum module size of 30 genes.

### Hub Genes and Key Genes Validation

Hub gene is defined as having high connectivity in a module and playing an important role in related clinical traits (Bai et al., 2021). Gene significance (GS) is defined as mediated *p*-value of each gene (GS = lgP) in the linear regression between gene expression and the clinical traits. Module membership (MM) is defined as the correlation between a given gene expression profile and a given model eigengene to represent intra connectivity. To identify hub genes in WGCNA, the threshold value of MM and GS was set to 0.8 and 0.2, respectively.

Hub genes in DEGs were constructed as the PPI network with the cut-off standard of interaction score >0.4 by the STRING database (http://www.string-db.org/) (Szklarczyk et al., 2021). Then, we visualized the PPI network by the Cytoscape software (version 3.6.1) (Le and Pham, 2017). In addition, we used the maximal clique centrality (MCC) computing method with the Cytohubba plugin to confirm the top 30 hub genes in the PPI network (Chin et al., 2014). Key genes were obtained by the intersection of hub genes in DEG-PPI network and selected modules eventually.

## Results

### Identification of Differentially Expressed Genes

**FIGURE 1:**
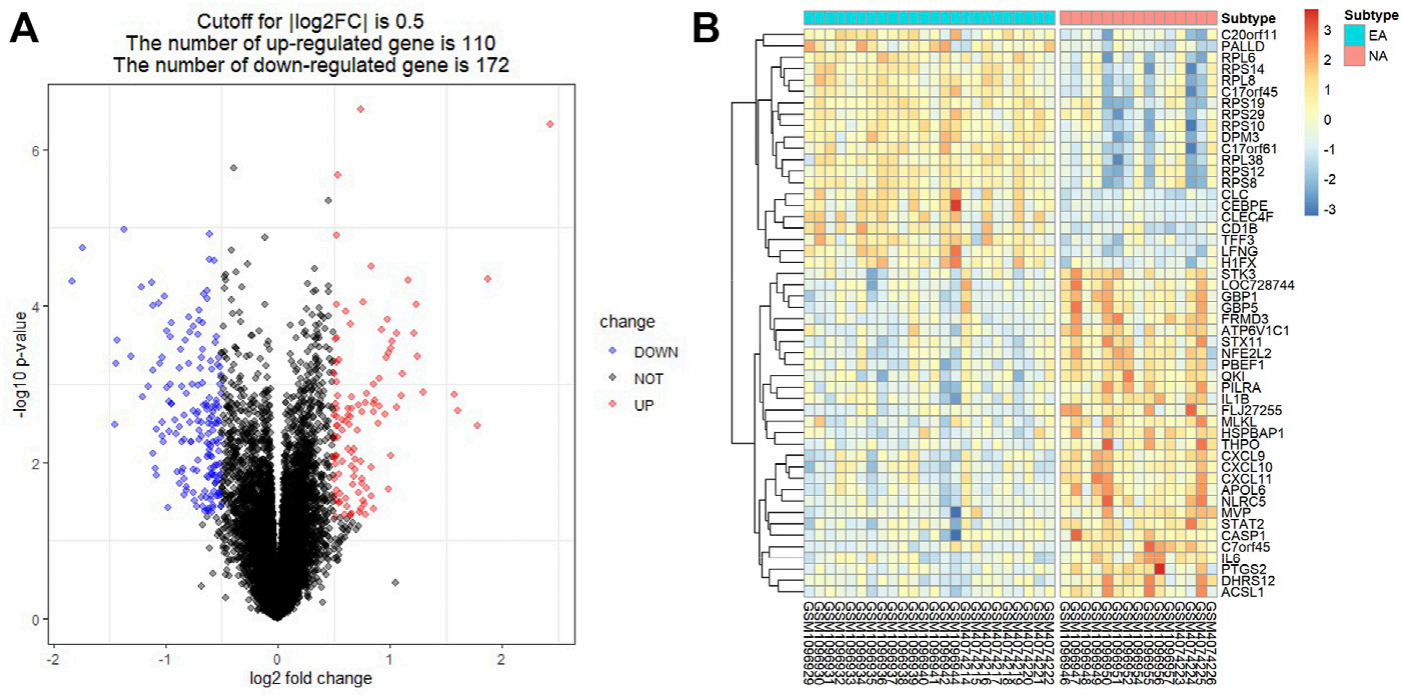
Identification of differentially expressed genes (DEGs) in induced sputum from eosinophilic asthma (EA) compared to those from neutrophilic asthma (NA). **(A)** Volcano plots showed the DEGs by the criteria of |logFC| > 0.5 and *p* < 0.05. The up-regulated genes were marked in red, while the down-regulated genes were marked in blue. **(B)** The top 50 DEGs with the largest logFC are shown in the heatmap.

To identify DEGs in induced sputum samples from EA patients compared to those from NA patients, expression profiles of GSE45111 and GSE137268 were downloaded from the GEO database. After data consolidation and removing the batch effects of the two microarray datasets, 282 DEGs (110 up-regulated and 172 down-regulated in EA patients) were screened out using the Limma package (*p* < 0.05, |logFC| > 0.5) (Supplementary Table S1). The result was shown with the volcano plot in Figure 1A. In addition, the logarithmic fold changes of the top 50 DEGs were showed in heatmap (Figure 1B).

### Functional and Pathway Enrichment Analyses for Differentially Expressed Genes

**FIGURE 2:**
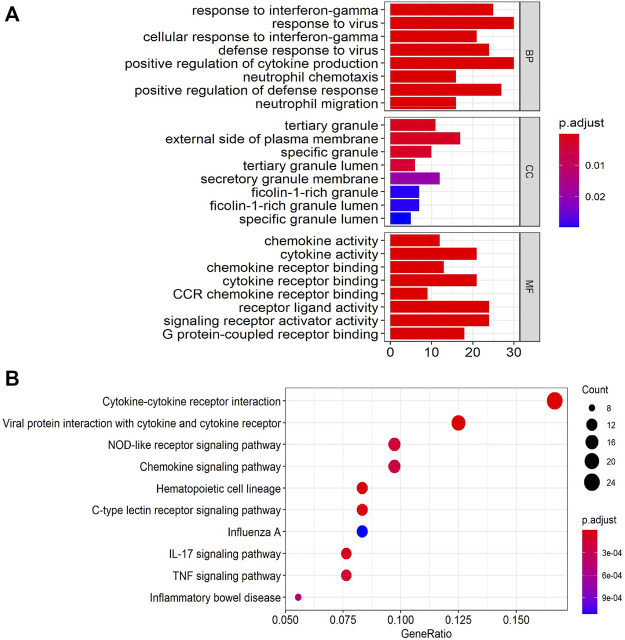
Gene Ontology (GO) and Kyoto Encyclopedia of Genes and Genomes (KEGG)enrichment annotations of the DEGs. **(A)** The results of GO enrichment categories included biological process (BP), cellular component (CC), and molecular function (MF). **(B)** The results of KEGG pathway enrichment analyses of the DEGs.

The enriched GO and KEGG analyses of the 282 DEGs were performed and visualized with the clusterProfiler package in R software. The biological process (BP) of GO enrichment analysis included response to interferon-γ (INFγ), response to virus and cellular response to INFγ. For the cellular component (CC), DEGs were enriched in tertiary granule, external side of plasma membrane, and specific granule. For the molecular function (MF), DEGs were significantly enriched in cytokine activity, chemokine activity, and cytokine receptor binding (Figure 2A). The result of GO enrichment of ascending and descending DEGs were shown in Supplementary Figures S1A,B, respectively. In addition, integrated DEGs were strongly involved in cytokine-cytokine receptor interaction, viral protein interaction with cytokine and cytokine receptor, and NOD-like receptor signaling pathway in KEGG pathway analysis (Figure 2B). The outcomes of KEGG pathway enrichment of ascending and descending DEGs were shown in Supplementary Figures S1C,D, respectively.

### Gene set enrichment analysi

**FIGURE 3:**
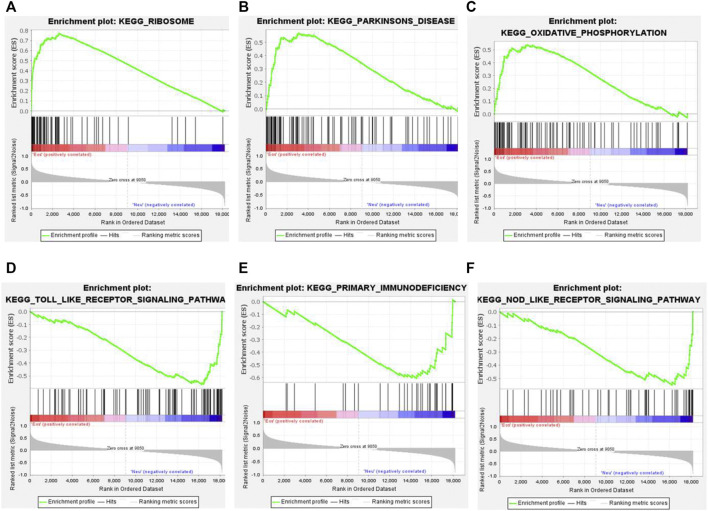
Gene set enrichment analysis (GSEA) plots of the most enriched gene sets in the EA and NA group. **(A–C)** The top 3 most enriched pathways in the EA group: ribosome **(A)**; Parkinson’s disease **(B)**; oxidative phosphorylation **(C)**. **(D–F)** The top 3 most enriched pathways in the NA group: toll-like receptor significant pathway **(D)**; primary immunodeficiency **(E)**; NOD-like receptor signaling pathway **(F)**.

The GSEA analysis was performed to identify unique pathways involved in the pathogenesis of EA or NA. The pathways related to ribosome, Parkinson’s disease, and oxidative phosphorylation were most significantly enriched in EA group. Toll-like receptor signaling pathway, primary immunodeficiency, and NOD-like receptor signaling pathways were most strongly enriched in NA group (Figures 3A–E).

### Weighted Gene Co-Expression Network Analysis

**FIGURE 4:**
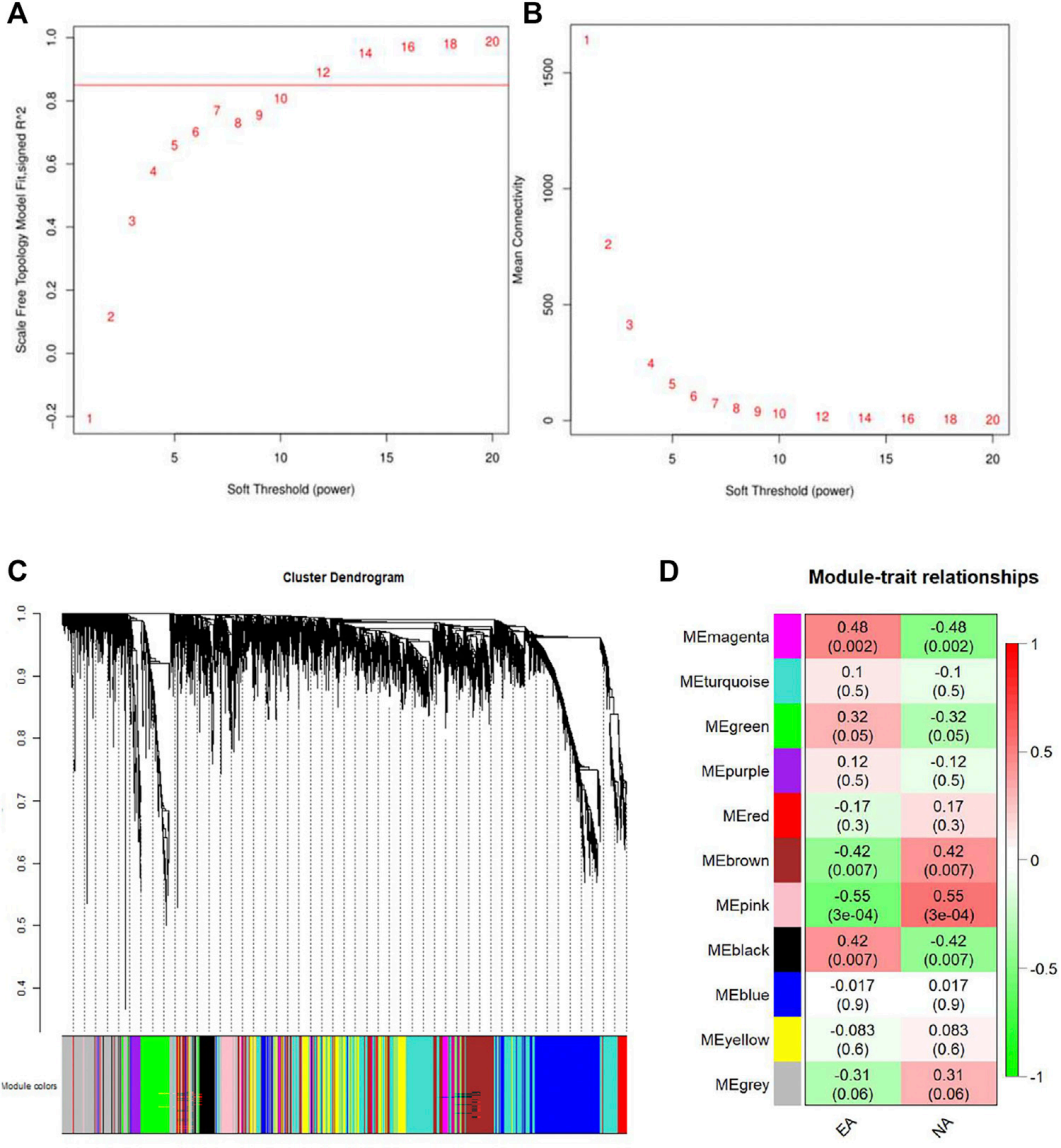
Identification of the gene modules related to EA and NA by weighted gene co-expression network analysis (WGCNA). **(A)** T scale-free fit index of soft-thresholding power. **(B)** Mean connectivity of various soft-thresholding powers. **(C)** Hierarchical clustering dendrograms of identified co-expressed genes were classified into different gene modules. Diverse colors reflected corresponding modules, and the grey module represented genes were not assigned to each network. **(D)** The heatmap of the relationship between each gene module and each subtype of asthma. The red represents positive correlation, while the green represents negative correlation.

WGCNA was performed to get a deeper insight into the association between the key modules and different asthma subtypes. As was shown in Figures 4A,B, the optimal soft-thresholding power was 12 if the correlational coefficient was >0.85. Eleven modules were generated via the average-linkage hierarchical clustering method (Figure 4C). The heatmap exhibited the correlation between different modules and subtypes of asthma. The magenta module was strongly positively correlated with EA group, so was the pink module with NA group (Figure 4D).

### Enrichment Analyses of Module Genes Identified by Weighted Gene Co-Expression Network Analysis

**FIGURE 5:**
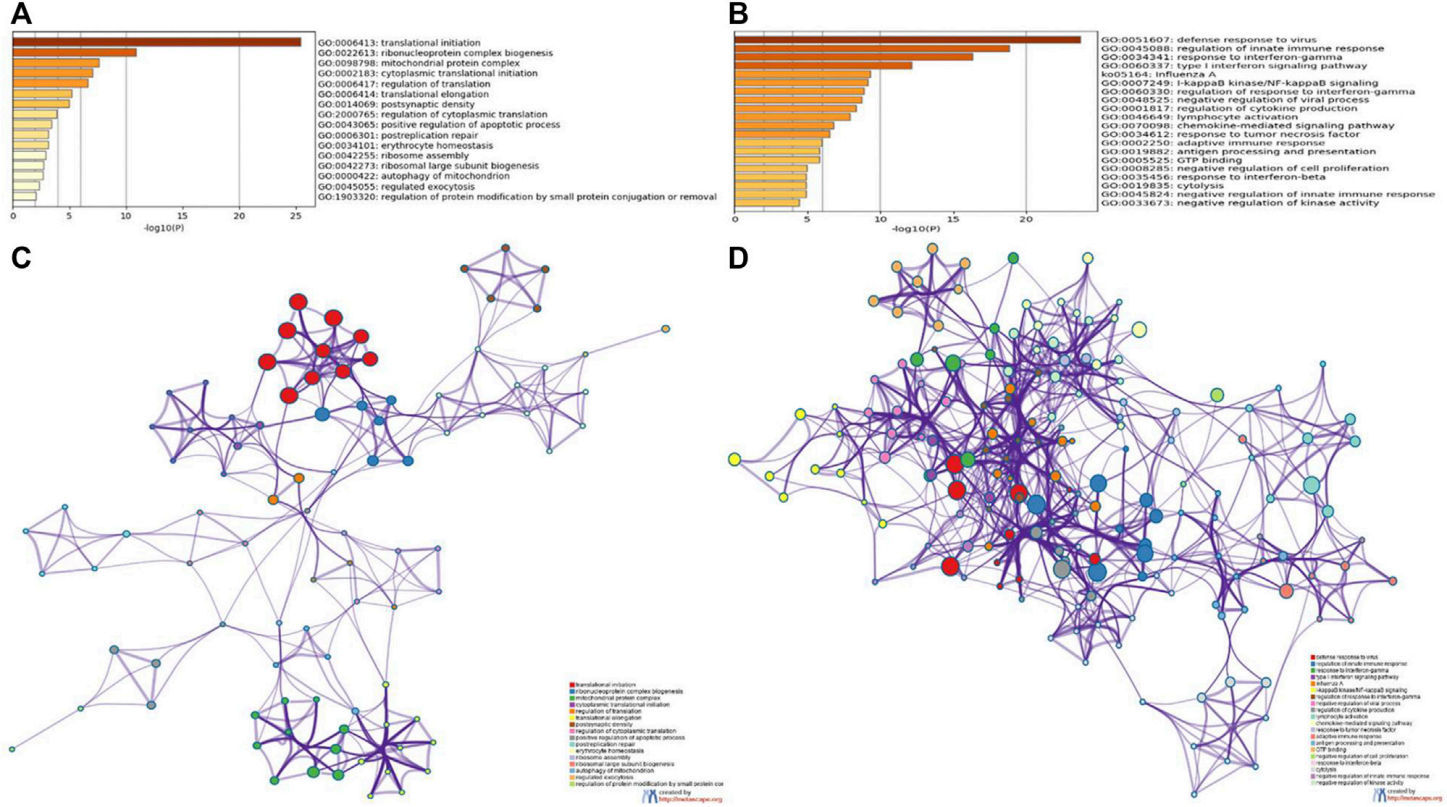
GO and KEGG enrichment analyses of module genes recognized by the WGCNA analysis. Bar plots of the GO and KEGG enriched terms colored by *p*-values in magenta module **(A)** and pink module **(B)**. Network of GO and KEGG enriched terms colored by genes in magenta module **(C)** and pink module **(D)**.

To further analyze the feature of the module genes, GO annotation and KEGG pathway enrichment analyses were performed using Metascape. As was shown in the bar graph and network plot (Figures 5A,C), genes in the magenta module were mainly involved in translational initiation, ribonucleoprotein complex biogenesis, mitochondrial protein complex, cytoplasmic translational initiation, and regulation of translation. The significantly enriched entries for pink module were defense response to virus, regulation of innate response, response to INFγ, and so on (Figures 5B,D).

### The Identification of Hub Genes and Key Genes

In the magenta and pink modules, there was positive correlation between MM and GS (Supplementary Figures S2A,B). Under the criterial of MM ≥ 0.8 and GS ≥ 0.2, 139 and 45 hub genes were recognized in magenta and pink genes, respectively (Supplementary Table S2).

**FIGURE 6:**
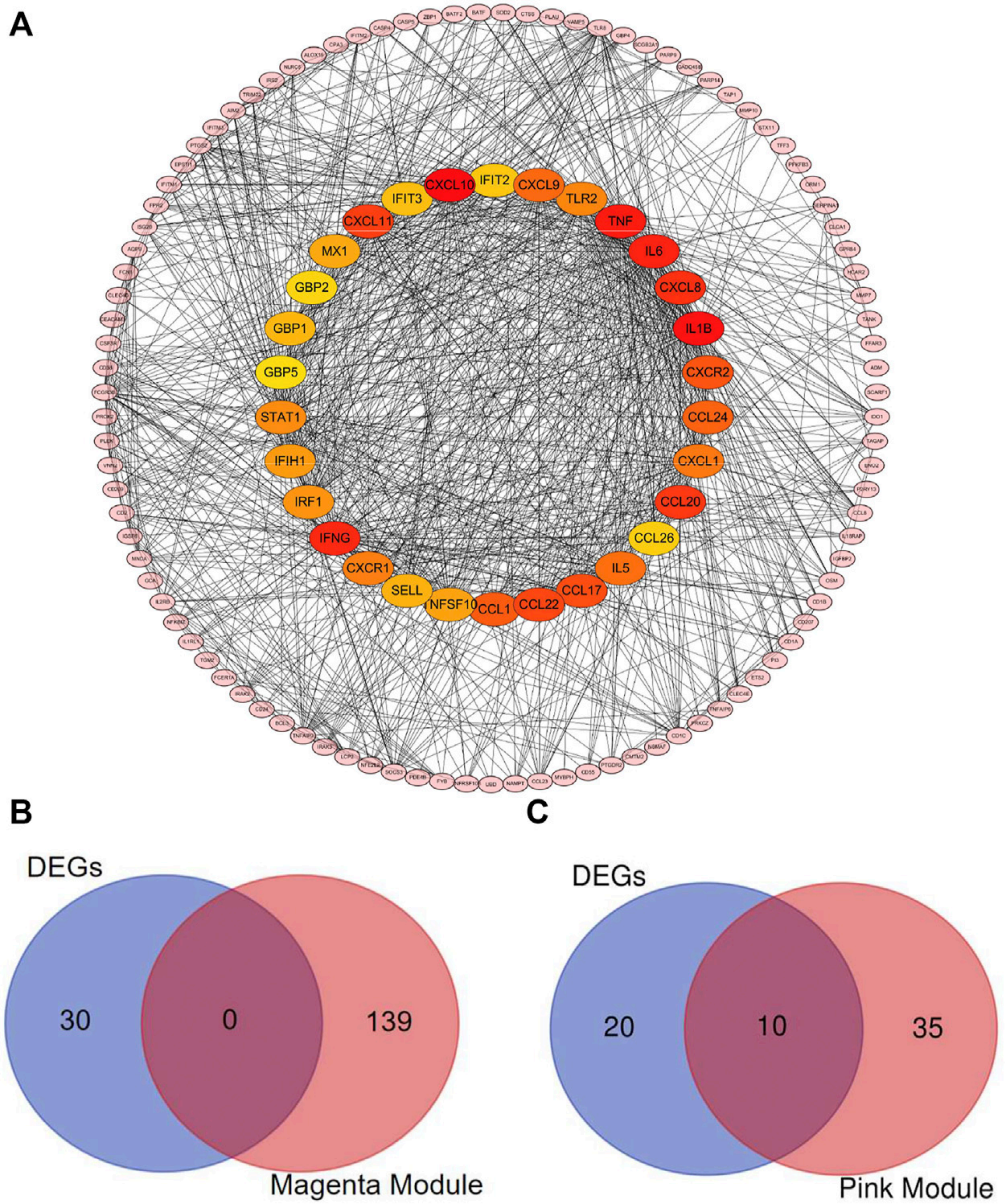
Identification of the hub genes and the key genes. The PPI network of the DEGs. **(A)** Top 30 hub genes in DEGs were confirmed with the Cytohubba plugin. The colors of the nodes reflect the degree of connectivity. The key genes are defined as the hub genes identified by both of DEG-PPI network and WGCNA method. **(B)** The hub genes in DEGs and in magenta module were shown using Venn diagram. No key genes were identified. **(C)** The hub genes in DEGs and in pink module were shown using Venn diagram. Ten key genes were identified.

To identify the hub genes in DEGs, the PPI network, which was constructed by STRING database, was visualized by the Cytoscape software. The network of DEGs included 129 nodes and 1,054 edges (Figure 6A). The top 30 hub genes in DEGs were confirmed by the MCC method with the Cytohubba plugin. To further filtrate the significant genes, we selected the overlapped genes that were identified by hub genes in DEG-PPI network and WGCNA as key genes (Figures 6B,C). The detailed information of 10 key genes (*IRFG*, *IRF1*, *STAT1*, *IFIH1*, *IFIT3*, *GBP1*, *GBP5*, *IFIT2*, *CXCL9,* and *CXCL11*) were presented in Table 1, including 10 genes in pink module.

**TABLE 1:** The information of 10 key genes.

Symbols	Full name	logFC	*P*. Value	Change	MM	GS	Module
IFNG	Interferon Gamma	−0.50256	0.000762	Down	0.805183	0.50692	Pink
IRF1	Interferon regulatory factor 1	−0.94617	0.0001660	Down	0.807654	0.55646	Pink
IFIH1	Interferon induced with helicase C domain 1	−0.71293	0.000234	Down	0.904381	0.54597	Pink
GBP1	Guanylate binding protein 1	−1.36625	1.06E-05	Down	0.947779	0.63034	Pink
GBP5	Guanylate binding protein 5	−1.06032	9.18E-05	Down	0.923995	0.57398	Pink
CXCL9	C-X-C motif chemokine ligand 9	−1.74202	1.80E-05	Down	0.815577	0.61743	Pink
CXCL11	C-X-C motif chemokine ligand 11	−1.21216	5.82E-05	Down	0.837962	0.58677	Pink
STAT1	Signal transducer and activator of transcription 1	−0.86358	0.000366	Down	0.837908	0.53135	Pink
IFIT3	Interferon induced protein with tetratricopeptide repeats 3	−0.51193	0.010624	Down	0.884857	0.39619	Pink
IFIT2	Interferon induced protein with tetratricopeptide repeats 2	−0.96638	0.001695	Down	0.868548	0.47634	Pink

10 key genes were obtained from intersection of hubs genes in DEGs, and hub genes in magenta and pink module, which included 10 down-regulated genes in EA, group.

DEGs, differentially expressed genes; EA, eosinophilic asthma; NA, neutrophilic asthma.

## Discussion

Airway neutrophilia is associated with asthma severity and poor responsiveness to steroid treatment (Ray and Kolls, 2017). In the present study, 282 DEGs in induced sputum samples were identified between patients with EA and NA, including 110 up-regulated genes and 172 down-regulated genes. Next, enrichment analyses, including GO, KEGG, and GSEA, were performed to explore the functions and signaling pathways related to the DEGs. After the construction of PPI network, the top 30 hub genes of DEGs were selected according to the Cytohubba algorithm. Using WGCNA analysis, pink and magenta modules were found to be strongly positively correlated with the EA and NA subsets, respectively. Finally, we defined 10 key genes according to intersection of the hub genes in DEGs and modules.

We performed enrichment analyses to explore the role of the DEGs. The significantly enriched entries for GO annotation demonstrated that DEGs were enriched in response to INFγ and cellular response to INFγ. INFγ is a Th1 cytokine that inhibits or reverses the allergic inflammation and therefore antagonizes the activating effects of Th2 cytokines including IL-4 and IL-13 in a variety of cell types (Davoodi et al., 2012). EA is orchestrated by Th2 cytokines, whereas NA is triggered by Th1 and Th17 cytokines (Erle and Sheppard, 2014; Trejo Bittar et al., 2015). This may explain the enrichment of DEGs in INFγ signaling pathway.

The GSEA data suggested that ribosome, Parkinson’s disease, and oxidative phosphorylation were mainly enriched in EA patients. Several epidemiological studies have implied that ribosome-inactivating stress are related to human mucosal epithelial illnesses (Moon, 2014). Moreover, it has been reported that intranasal neutrophilic rhinitis can be triggered by some of ribosome-inactivating trichothecenes (Islam et al., 2007; Corps et al., 2010; Carey et al., 2012). Both NA and neutrophilic rhinitis are characterized by neutrophilic inflammation. Therefore, NA subset may develop ribosomal inactivation leading to ribosome pathway enriched in EA group. Cheng’s study suggested that patients with asthma had a higher risk of developing Parkinson’s disease in their later life (Bower et al., 2006; Cheng et al., 2015). The relation between the pathogenesis of Parkinson’s disease and asthma requires further study. Toll-like receptor signaling pathway, primary immunodeficiency, and NOD-like receptor signaling pathway were mainly enriched in NA subset. Toll-like receptors (TLRs) belong to pattern recognition receptors, which play an important role in the recognition of pathogens (Xiao et al., 2012). TLRs can affect epithelial and immune cell function in asthma (Mishra et al., 2018). Of note, TLR4 is essential for Th17-driven neutrophilic airway inflammation and neutrophil recruitment (McAlees et al., 2015; Wan et al., 2020). NOD-like receptors (NLRs) are a relatively new member of the pattern recognition receptor superfamily (Song and Li, 2018). They are the key players in the innate immune responses of inflammatory lung diseases (Chaput et al., 2013). Several studies have demonstrated that NLRP3 plays an important role in asthma (Birrell and Eltom, 2011; Simpson et al., 2014; Kim et al., 2017; Chen et al., 2019). Chen and colleagues reported that blockade of the NLRP3/caspase-1 axis prevented the progression of TDI-induced NA (Chen et al., 2019).

To further investigate the relationship between co-expression genes and different asthma subtypes, we performed WGCNA. Eleven modules were defined and magenta and pink modules were most significantly positively correlated with EA and NA group, respectively. The result of the enrichment for the magenta module included translational initiation, ribonucleoprotein complex biogenesis, mitochondrial protein complex, cytoplasmic translational initiation, and regulation of translation. The result of the enrichment for the magenta module included defense response to virus, regulation of innate response, response to IFNγ, and so on. Viral infection is a common trigger for the exacerbation of asthma (Mikhail and Grayson, 2019). Neutrophils are considered to play a pivotal role in the interplay between viral infection and asthma exacerbation (Holtzman, 2012). Our data support the connection among viral infections, asthma exacerbation and NA.

After taking intersection of the hub genes in DEGs and the modules, 10 keys genes were identified, including *IRFG*, *IRF1*, *STAT1*, *IFIH1*, *IFIT3*, *GBP1*, *GBP5*, *IFIT2*, *CXCL9,* and *CXCL11*. IFNγ (*IRFG*) is a member of type II interferon involved in the chemotaxis of human neutrophils by up-regulating the expression of neutrophils chemokine receptors *CCR1* and *CCR3* (Bonecchi et al., 1999). Recent studies have revealed that IFNγ could up-regulate and down-regulate the expression of *CXCL10* and *SLPI*, respectively, which further resulted in increased AHR and steroid resistance in severe asthma (Raundhal et al., 2015; Oriss et al., 2017). Interferon regulatory factor 1 (*IRF1*) is involved in a series of pathophysiological processes in viral infection, tumor immune surveillance, and proinflammatory injury (Wang et al., 2020). Increased IRF1 expression is implicated in reduced responsiveness to glucocorticoids (Tliba et al., 2008; Chapin et al., 2015). Glucocorticoids induce *DUSP1* expression and downregulates MAPK activity, thereby inhibiting inflammatory response (Newton et al., 2017). However, increased *DUSP1* expression also increases the activity of IRF1 and IRF1-dependent genes, including *CXCL10* (Shah et al., 2016). *CXCL10* can promote airway inflammation and hyperresponsiveness, and virus-induced exacerbation of asthma, resulting in poor response to glucocorticoids (Medoff et al., 2002; Wark et al., 2007). In conclusion, *IRF1* could be a key gene for glucocorticoids resistance of NA. Signal transducer and activator of transcription 1 (*STAT1*) regulates production of Th1 cell-specific cytokine to alter inflammatory response and it is a key mediator in IFNγ signaling (Chen et al., 2017; Zhang et al., 2018). Several studies have shown that STAT1 participants in the differentiation of Th17 cells and it may become a key gene in the development of NA (Villarino et al., 2010; Kimura et al., 2008; Nielsen et al., 2015). Interferon induced with helicase C domain 1 (*IFIH1*) encodes MDA5, which is an intracellular sensor of viral RNA that triggers an innate immune response and is associated with the production of type I interferon and proinflammatory cytokines (Zhang et al., 2011; Mibayashi et al., 2007; Pichlmair et al., 2009). Guanylate binding protein 1 (*GBP1*) and guanylate binding protein 5 (*GBP5*) are two member of the *GBP* family and mediate cellular response to IFNγ in infection and inflammation (Tripal et al., 2007; Britzen-Laurent et al., 2010; Li et al., 2020). Interferon induced protein with tetratricopeptide repeats 3 (*IFIT3*) and interferon induced protein with tetratricopeptide repeats 2 (*IFIT2*) are both members of *IFITs* and are highly expressed in the innate immune response of cells to viral infection (Fleith et al., 2018). C-X-C motif chemokine ligand 9 (*CXCL9*) and C-X-C motif chemokine ligand 11 (*CXCL11*), ligands of chemokine receptor *CXCR3*, are induced by IFNγ (Proost et al., 2004; Tworek et al., 2013). These are especially involved in Th1-type response and correlates with the tissue infiltration of T cells (Koper et al., 2018). Southworth found that higher expression of *CXCL11* appeared in moderate asthma after rhinovirus infection (Southworth et al., 2020). Ghebre reported that sputum *CXCL9* level and serum *CXCL11* level increased during asthma exacerbation (Ghebre et al., 2019).

It is intriguing to notice that IFN-stimulated genes were highly enriched in the pink module, which indicated the significant role of interferon in the pathogenesis of NA. A recent study demonstrated that IFN-stimulated genes expression is increased in severe asthma (Bhakta et al., 2018). In addition, Silva’s study revealed the overexpression of ISGs in sputum from NA (da Silva et al., 2017). The interferon family consists of three kinds of interferons, namely type I interferon, type II interferon, and type III interferon (Negishi et al., 2018). Type II interferon (IFNγ) has been discussed above. Type I interferon (interferon-alpha/beta) and Type III interferon (interferon-lambda) play crucial roles in host defense against infectious agents (Hansel et al., 2013), thus inhibiting the exacerbation of asthma. Nevertheless, the role of type I interferon and type III interferon in NA has not been reported yet (da Silva et al., 2017; Rich et al., 2020).

There are several limitations in this work. First, we have not obtained valuable clinical data, especially induced sputum cell counts. Second, cut-off standard of logFC was not high enough so that some of the expression difference of key genes between EA and NA group was subtle. Third, this work had the low number of samples and lack of the validation.

In conclusion, we have identified several enriched pathways in the EA compared to the NA. By intersection of hub genes in DEGs and modules, 10 key genes were defined. These key genes may provide new insights into the pathogenesis of NA, and become potential therapeutic targets of NA.

## Data Availability

The datasets analyzed during the present study are available from the Gene Expression Omnibus repository (https://www.ncbi.nlm.nih.gov/geo/query/acc.cgi?acc=GSE45111 and https://www.ncbi.nlm.nih.gov/geo/query/acc.cgi?acc=GSE137268).

## Supplementary Material

Supplementary Figure S1 Function annotations analyses of DEGs. GO enrichment terms of up- and down-regulated genes are presented in **A, B**. KEGG pathway terms of up- and down-regulated genes are presented in **C, D**.

Supplementary Figure S2 A scatter plot of the GS for the EA or NA classification versus the MM in the magenta **(A)** or pink **(B)** modules; the correlation and *P*-value are under the title. EA, eosinophilic asthma; NA, neutrophilic asthma; GS, gene significance; MM, module membership.
