# Supplementary figures and images for: Identification of Key Signaling Pathways and Genes in Eosinophilic Asthma and Neutrophilic Asthma by Weighted Gene Co-Expression Network Analysis
Source: Front Mol Biosci. 2022 Feb 2;9:805570. doi: 10.3389/fmolb.2022.805570 (PMC8847715; doi:10.3389/fmolb.2022.805570)

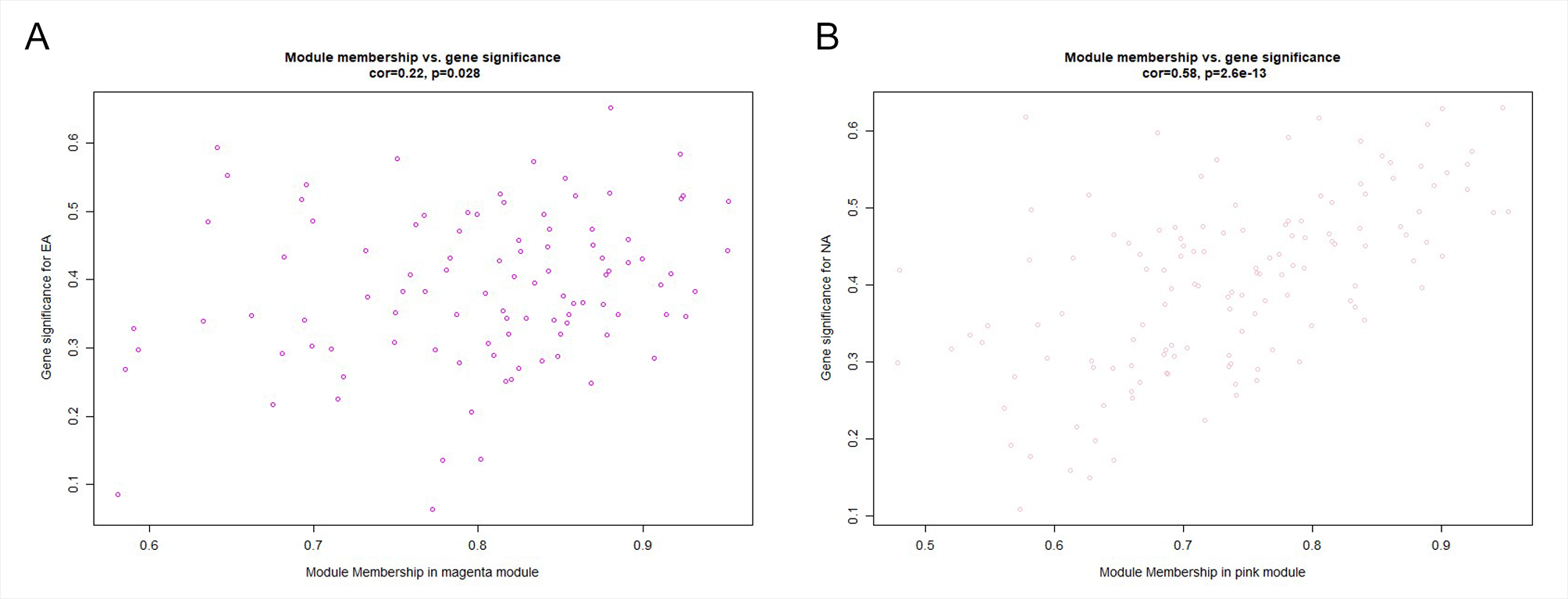

Supplement: Supplementary file 2 [file Image2.tif]

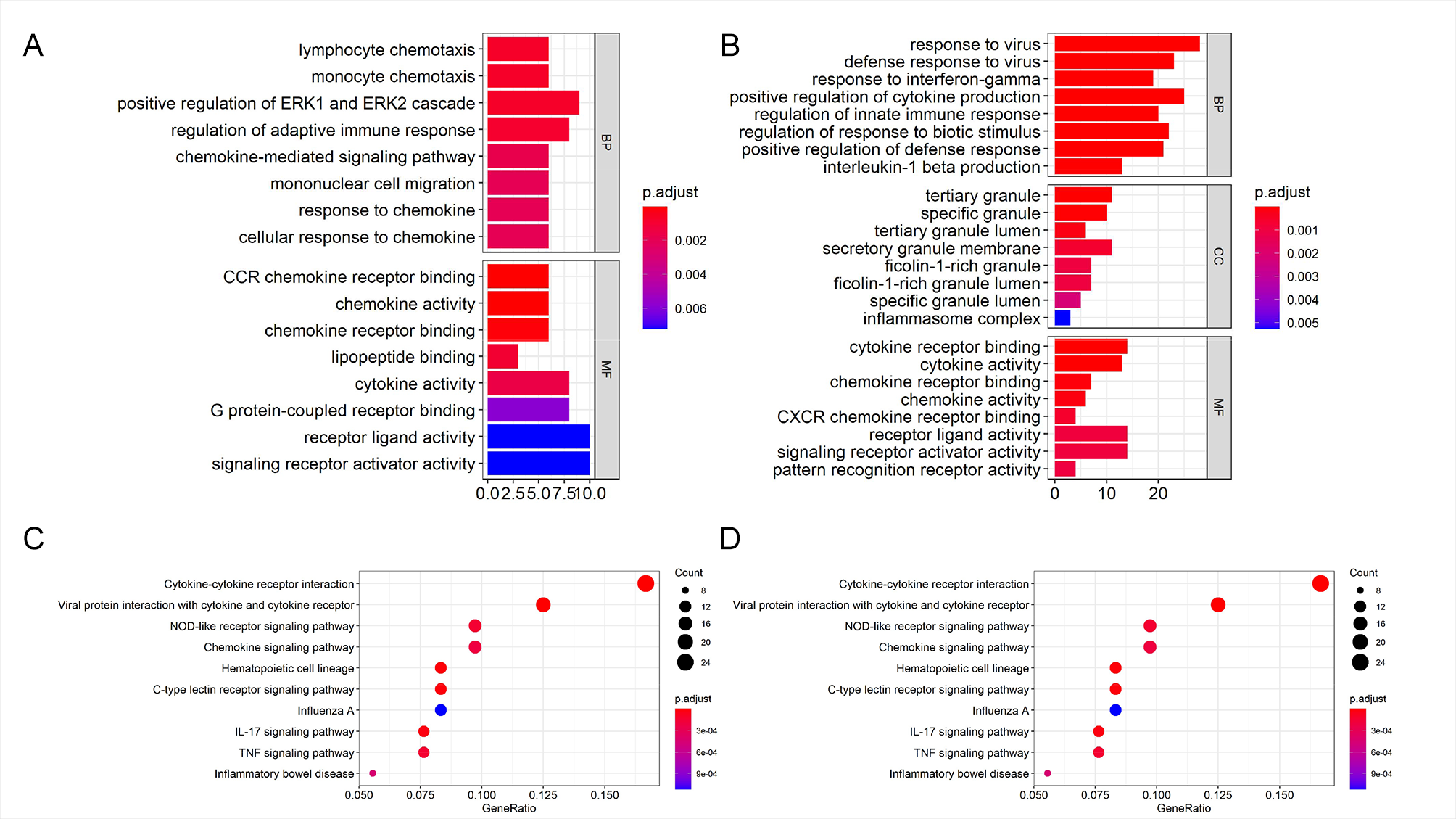

Supplement: Supplementary file 3 [file Image1.tif]
